# Scrotal tick damage as a cause of infertility in communal bulls in Moretele, South Africa

**DOI:** 10.4102/jsava.v90i0.1966

**Published:** 2019-10-23

**Authors:** Cheryl M.E. McCrindle, Masethe J. Maime, Ester A. Botha, Edward C. Webb, Mario P. Smuts

**Affiliations:** 1 Department of Agriculture and Animal Health, School of Agriculture and Life Sciences, University of South Africa, Pretoria, South Africa; 2 Department of Animal and Wildlife Sciences, Faculty of Agricultural and Natural Sciences, University of Pretoria, Pretoria, South Africa; 3 Department of Production Animal Studies, Faculty of Veterinary Science, University of Pretoria, Onderstepoort, South Africa

**Keywords:** bull fertility, communal farming systems, *Hyalomma rufipes*, *Amblyomma hebraeum*, calving rate, Moretele, North West Province

## Abstract

Calving rate in communal cattle influences both food security and socio-economics in rural households. A previous study indicated that scrotal damage caused by ticks could affect the fertility of communal bulls and reduce the annual calving rate. The objectives of the study were to investigate the annual calving rate in communal herds by counting calves during herd visits, perform breeding soundness examinations on bulls and identify adult ticks attached to their genitalia. This prospective longitudinal survey was based on participatory rural appraisal. Calving rates were estimated in cows (*n* = 2398) from 100 randomly selected communal herds in Moretele over 12 months in 2013, during routine visits by animal health technicians. Randomly selected bulls (*n* = 50) from these herds were tested for *Brucella abortus, Trichomonas foetus* and *Campylobacter fetus* subspecies venerealis. The calving rate was 35.86% (0.359). The mean scrotal circumference was 37.63 ± 3.42 cm. Total sperm motility was 78.73 ± 35.73%; progressive sperm motility was 27.39 ± 15.81% and non-progressive sperm motility was 51.34 ± 19.92%. Thirty-five of the 38 bulls examined for breeding soundness exhibited severe scrotal and preputial lesions caused by the adult ticks *Amblyomma hebraeum* and *Hyalomma rufipes*. Tick control methods used included spraying (*n* = 20), pour-on (*n* = 11), no control (*n* = 1) and various (*n* = 18). It was concluded that in Moretele genital tick damage had a more serious impact on the fertility of communal bulls than contagious diseases. Targeted acaricidal spot treatment of the genitalia of communal bulls to prevent infestation is recommended, as tick control strategies used by farmers appeared to be inadequate.

## Introduction

In areas of South Africa with low soil fertility and rainfall of less than 500 mm per year, commercial and communal extensive beef production systems are sustainable options (Grobler 2015; Musemwa et al. 2008). Communal livestock farming in South Africa focuses mainly on food security at household level rather than profitability (Mmbengwa et al. 2015). According to the Department of Agriculture Forestry and Fisheries website (DAFF 2019), the recommended calving percentage for beef cattle in South Africa is 85%. Calving percentage is the number of calves born from the number of female cattle served by a bull (Reiling 2011). The calving rate is defined as the percentage of mated (or inseminated) cows that calve per breeding season (Fahey 2002). Bull fertility is known to influence calving rates in communal cattle rearing systems in South Africa (Mokantla et al. 2004; Sekokotla 2004). Fertility in range bulls is assessed by using breeding soundness examinations (Alexander 2008; Chenoweth & McPherson 2016; Chenoweth, Spitzer & Hopkins 1992; Irons, Nothling & Bertschinger 2007). Irons et al. (2007) mentioned that the breeding soundness examination includes certification related to the purpose for which the bull is to be used – it is either certified (fertile) or not. Infectious diseases that could affect the calving rate in the study area are bovine brucellosis, bovine genital campylobacteriosis and bovine trichomoniasis (Mokantla et al. 2004).

*Amblyomma hebraeum* is a long-mouthed tick responsible for the transmission of heartwater (*Ehrlichia ruminantium*) in bushveld biomes in southeastern Africa (Steyn, McCrindle & Du Toit 2010). Adults feed on the hairless areas under the tail, on the perineum and around the genitalia of cattle, and can cause severe skin lesions with secondary miasis (Walker et al. 2003). *Hyalomma rufipes* is a long-mouthed tick known to transmit anaplasmosis (*Anaplasma marginale*) in cattle (De Waal 2000). It is widely distributed in Africa and is known to be carried by migratory birds. The adults of *H. rufipes* attach to the perianal area, perineum and genitalia of cattle. They are known to cause severe lesions and even abscesses at feeding sites (Walker et al. 2003). The seasonal prevalence of these ticks has been described by Rechav and De Jager (1991).

In South Africa, all acaricides used for livestock must be registered under the *Fertilizers, Farm Feeds, Agricultural Remedies and Stock Remedies Act*, 1947 (Act 36 of 1947). There are four major groups of acaricides registered for use in cattle in South Africa: amidines, organophosphates, pyrethroids and macrocytic lactones (Mekonnen 2005).

During a longitudinal study in Jericho, North West Province (NWP), Mokantla et al. (2004) found that 43% of cows sampled did not become pregnant over their 400-day study period. They suggested that sub-fertile bulls were the main reason for this, mainly because of tick damage to their genitalia. They did, however, admit that their sample size of 13 bulls was small. Unfortunately, no other published studies have, to our knowledge, addressed this problem.

The aim of this study was therefore to use a participatory farming systems approach to investigate the possible links between tick damage to genitalia and bull fertility in communal herds in the Moretele District, NWP.

## Materials and methods

### Study design

This was a non-experimental, inductive, observational research study with the collection of empirical categorical and numerical data to derive a theory and hypothesis. A prospective descriptive cross-sectional longitudinal study design (Centre for Evidence Based Medicine 2014) was used to survey the calving rate of 100 randomly sampled communally grazed cattle herds in the study area (Moretele District, NWP, South Africa). These herds were utilising communal grazing and a herd was defined as a group of cattle belonging to a particular owner. Census was routinely performed on all these herds by the state veterinary services (SVS) personnel and 75 bulls and 2398 breeding cows were counted at the beginning of the survey. Because of the nature of communal grazing, bulls ran with the cows and could therefore mate cows that did not belong to the bull’s owner (Maime 2016).

The conceptual framework of farming systems research is based on community participation combined with scientific investigation. Martin et al. (2018) have crystallised this concept as follows:

Comprehensive analysis of the sustainability transition provides perspectives on the interplay between resources, resource management, and related performances of farming systems on the one hand and technical, economic, and sociocultural dimensions of change on the other. (n.p.)

In this study, a systems approach was adopted that integrated direct scientific observations obtained during breeding soundness examinations (Chenoweth et al. 1992; Chenoweth & McPherson 2016), monthly visits to count cattle, identification of the tick species on genitalia of bulls and farmers’ opinions on current tick control strategies. Participatory workshops were arranged to enable stakeholders, end users and role-players to analyse, share and enhance their knowledge before and during the project and more specifically to plan, facilitate, manage and evaluate the examination of bulls and record the calving rate before and during the 12-month study period. Participatory workshops have been defined as participatory rural appraisal (PRA) or participatory learning and action (PLA). Chambers (2015) defined these concepts as follows:

Ideologically and epistemologically PRA/PLA seeks and embodies participatory ways to empower local and sub-ordinate people to examine and analyse their realities, to enhance and express their knowledge and to take action. It can be understood as having three main components: facilitators’ behaviours, attitudes and mind-sets linked with precepts for action; methods which combine visuals, tangibles and group activities; and sharing without boundaries. (p. 32)

### Setting

Moretele is part of the Bojanala District in the eastern region of the NWP, South Africa (DAFF NWP 2018). It is located approximately 60 km north of Pretoria, bordering the Limpopo and Gauteng Provinces, and consists of 66 villages and 10 farms. It is divided into three veterinary service delivery wards, each managed by a chief animal health technician (AHT) who executes regulatory animal health activities and provides support for the responsible state veterinarian (Maime 2016).

### Study population and sampling strategy

Ten villages out of 66 in the Moretele District municipality, NWP, were randomly selected to participate in the study. The villages included Mathibestad, Kgomokgomo, Tladistad, Mmatllwaela, Sutelong, Bollantlokwe, Lebalangwa, Mmakaunyane, Mootla and Ratjiepane. In each village, 10 communal farmers (five owning ≥ one bull and five without) were randomly selected resulting in a total of 100 farmers. Inclusion and exclusion criteria ware based on:

- Only farmers who volunteered to participate in the research study.
- Five out of 10 farmers selected per village had to own a bull and a minimum of 10 breeding cows, and the bull or bulls had to be 2 years and older.

A total of 100 herds that included both breeding bulls and cows were used as the study population. The sampling strategy for estimating the calving rate was to be determined by AHT regularly visiting all 100 herds over a 12-month period in 2013 while physically counting all cows and calves born over the study period, as well as observing the bulls. For the purposes of the study, as these were range cattle, an adult cow was defined as older than 36 months, or that had already calved; a heifer was defined as a cow less than 36 months old that had not yet calved. All adult cows (*n* = 2398) were included at the beginning of the survey. The herds included 75 bulls of which 50 were randomly selected for inclusion in breeding soundness evaluations. During the initial participatory workshop, communal farmers were consulted on the best times to make appointments to count the cattle and test the bulls. Prior to identification of selected bulls with ear tags, the condition of cattle handling facilities or crush pens in all 10 villages was evaluated. Crush pens were upgraded by farmers, with assistance from AHT employed by the SVS to ensure suitability for semen collection and breeding soundness evaluations.

### Interventions

Apart from testing for infectious diseases that could affect fertility (bovine brucellosis, genital campylobacteriosis and trichomoniasis) and breeding soundness examinations in range bulls (Maime 2016; Mokantla et al. 2004), there were no other interventions.

### Data collection

Qualitative data (such as the methods they used for controlling ticks on their bulls) (Table 3) were collected from participatory workshops, observations and interviews as previously described. Quantitative data were collected from herd surveys (numbers of cows and calves over 12 months), disease testing and breeding soundness examination of bulls and identification of ticks found on the genitalia of bulls. Details of data collected included the following:

- Herd survey data by AHT who work regularly with the communal farmers and perform an annual census of their herds. There were consequently no language or cultural barriers to data collection.
- All bulls were tested for transmissible venereal diseases caused by *Brucella abortus*.
- Bulls were tested for *Trichomonas foetus* and *Campylobacter fetus* as described in detail by Maime (2016). This yielded binomial (dichotomous) categorical data.
- Data on breeding soundness of bulls were collected by individual clinical examination, body condition scoring and palpation of the genitalia (Chenoweth 1994; Chenoweth & McPherson 2016; Maime 2016).
- Assessment of semen quality was performed as described by Maime (2016). In short, semen from 38 bulls was collected by means of electro-ejaculation. Immediately following collection, macroscopic data (colour, pH and volume) of each ejaculate were recorded and a morphology smear was prepared by using an Eosin-Nigrosin stain. Computer-assisted sperm analysis (CASA) was performed by using the Sperm Class Analyser^®^ system (SCA^®^ – Microptic, Barcelona, Spain) to evaluate the percentage (%) of total motile, progressively motile and rapidly motile sperm. In addition, the average path, curvilinear and straight-line velocities as well as the amplitude of lateral head displacement of sperm were determined. Sperm concentration (haemacytometer) and morphology were evaluated on the day of collection at the semen laboratory of the Faculty of Veterinary Science, University of Pretoria, Onderstepoort.
- Adult ticks feeding on the scrotum and prepuce of bulls in the project were photographed and specimens collected were identified according to their morphology by SVS personnel.

### Data analysis

Observational statistics were used to estimate the calving rate by recording the number of births in communal cows over the 12-month study period. Data on semen quality, clinical examination, scrotal circumference (SC) and damage to external genitalia were analysed using observational statistics to estimate the breeding soundness of bulls. Microsoft Excel Version 15.1 (Microsoft Corporation, United States) was used for compiling data before it was transferred to SPSS 20 computer software for data summary.

### Ethical considerations

All cattle owners and respondents signed a letter of consent and ethical approval was granted by the University of Pretoria ethics committee.

The research protocol for this research and informed consent forms were approved by the Research Committee of the Department of Animal and Wildlife Sciences in 2011.

## Results

Bulls on the communal grazing area in the study ran with the cows throughout the year. The body condition score was 3 or more for all the bulls in the study over the entire study period. Over the 12-month study period in 2013, 860 calves were born to the 2398 adult cows and 75 bulls counted at the beginning of the survey resulting in a calving rate of 35.86%. Two (4%) of the randomly selected 50 bulls tested positive for *B. abortus* and were culled before the study began.

One bull tested positive for *T. foetus* from the first sheath scrapings collected. However, three subsequent samples from the same bull tested negative and although the result was regarded as a potential false positive, the bull was withdrawn from the study. During the study, nine other bulls were withdrawn by their owners before the breeding soundness evaluation could be done. The remaining 38 tested negative for *T. foetus* and *C. fetus*. All bulls (100%) tested negative for *C. fetus*.

The SC measurement (Chenoweth 1994; Chenoweth et al. 1992) from the bulls included in the study was 37.63 cm (Table 1).

**TABLE 1:** Scrotal measurements of bulls (*n =* 38) per breed during breeding soundness evaluation (Maime 2016).

Variables	Breed		
	Brahman	Brahman cross-bred	Tuli
Number of bulls	24	13	1
Range of scrotal circumference (cm)	32–42	30–44	39
Mean scrotal circumference	37.92	37	39
Scrotal circumference/breed	2.92	4.36	0
Standard error	0.60	1.21	0
Median	38	38	39
Mode	37	39	0
Sample variance	8.51	19	0
Kurtosis	−0.40	−0.64	0
Skewness	−0.43	0.11	0
Sum	910	481	39
Confidence level (95.0%)	1.23	2.63	0
Number of bulls < 34 cm	2	3	0
(%) Bulls < 34 cm	8	23	0

The age and breed of bulls (*n* = 38) examined for breeding soundness, with observations on sperm motility and morphology in each bull, is shown in Table 2.

**TABLE 2:** Results of breeding soundness examinations and semen evaluation done on communal bulls.

Breed	Tick damage (Yes/No)	Age (years)	Sperm motility			Sperm morphology					Good quality (Yes/No)
			TM	PM	NPM	LN	DN	Head Abn	Mid P Abn	Tail Abn	
Brahman	No	5	95.2	57.9	37.3	64	10	2	3	21	Yes
Brahman	Yes	5	93.2	12.3	80.9	50	0	5	2	43	No
Brahman X	Yes	3	86.1	27.6	58.5	54	0	2	12	32	No
Brahman X	Yes	4	71.0	36.6	34.4	10	10	25	40	15	No
Brahman X	Yes	3	75.5	14.6	60.8	Very	Little	Sperm	-	-	No
Brahman	Yes	4	0.0	0.0	0.0	Very	Little	Sperm	-	-	No
Brahman	Yes	5	54.8	37.8	17.1	Very	Little	Sperm	-	-	No
Brahman	No	4	88.7	60.6	28.0	50	20	5	10	15	Yes
Brahman X	Yes	4	98.9	22.4	76.4	28	17	0	10	45	No
Brahman	Yes	3	98.0	20.3	77.7	14	43	3	28	12	No
Brahman	Yes	2	98.0	33.6	64.4	15	34	4	9	38	No
Brahman X	Yes	3	90.3	20.8	69.5	15	37	6	3	39	No
Brahman X	Yes	4	93.6	46.8	46.8	36	5	9	3	47	No
Brahman X	Yes	3	37.3	6.0	31.3	29	6	11	9	45	No
Brahman	Yes	3	90.6	38.9	51.6	17	9	1	36	37	No
Brahman	Yes	7	60.7	11.7	49.0	37	0	7	19	37	No
Brahman	Yes	4	53.4	15.8	37.6	37	7	2	10	44	No
Brahman	Yes	5	85.8	14.2	71.6	56	14	6	2	22	No
Brahman	Yes	3	95.0	16.3	78.7	61	8	2	2	27	No
Brahman X	Yes	4	7.3	5.2	2.1	23	32	8	3	34	No
Brahman X	Yes	3	58.3	11.7	46.7	36	14	9	9	32	No
Brahman	Yes	3.5	82.5	22.8	59.7	16	20	10	7	47	No
Brahman	Yes	5.5	29.1	.0	29.1	0	21	14	3	62	No
Brahman	Yes	5	59.3	16.2	43.1	20	10	32	30	8	No
Brahman	Yes	3	96.5	34.8	61.7	43	13	10	0	34	No
Brahman	Yes	3	82.6	24.2	58.4	79	9	4	1	7	No
Tuli	Yes	4.5	92.2	13.0	79.3	92	0	0	2	6	No
Brahman X	Yes	4	98.3	48.1	50.3	62	4	2	8	24	No
Brahman	No	3	95.7	50.3	45.5	85	1	2	0	12	Yes
Brahman	Yes	3.5	100.0	41.4	58.6	44	17	4	6	29	No
Brahman X	Yes	4	97.5	39.7	57.8	54	7	6	5	28	No
Brahman X	Yes	3	94.8	27.6	67.3	61	9	4	1	25	No
Brahman	Yes	4	94.3	24.4	69.8	49	24	2	0	25	No
Brahman X	Yes	5	89.8	46.3	43.5	36	20	20	2	22	No
Brahman	Yes	2.5	97.9	41.5	56.4	44	27	2	3	24	No
Brahman	Yes	4	71.7	27.2	44.4	53	3	3	2	39	No
Brahman	Yes	4	84.7	45.1	39.6	20	6	5	1	68	No
Brahman	Yes	5	93.4	27.2	66.2	74	3	1	0	22	No

SC, scrotal circumference; TM, total motility; PM, progressive motility; NPM, non-progressive motility; LN, live normal morphological sperm; DN, dead normal morphological sperm; Head Abn, head abnormalities; Mid P Abn, mid-piece abnormalities; Tail Abn, tail abnormalities; X, cross-bred.

**FIGURE 1:**
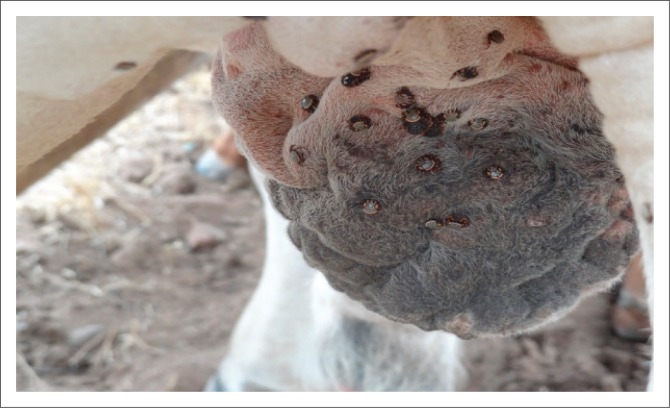
Adult long-mouthed ticks of the species *Hyalomma rufipes* and *Amblyomma hebraeum* attached to the scrotum.

Methods used by the farmers for the control of tick infestations on bulls are presented in Table 3. Three bulls showed no signs of any scrotal damage typical of tick infestation; the other 35 bulls showed scrotal and preputial abscesses and thickening of the scrotal skin together with nodules caused by infestation with long-mouth ticks. According to the owners, these three bulls were only recently introduced to the herd. Adult ticks found feeding on bulls in this study (Figure 1) were identified as *A. hebraeum* and *H. rufipes*.

**TABLE 3:** Methods used by farmers for controlling ticks on their communal bulls (*n* = 50).

Method of tick control	Frequency (*n* = 50)	Proportion
Plunge dip and hand spray	1	0.02
Plunge dip, pour-on and injectable	1	0.02
Spray race only	20	0.4
Spray race and pour-on	4	0.08
Spray race, pour-on, hand dressing and injectable	1	0.02
Spray race, pour-on and injectable	2	0.04
Spray race and injectable	1	0.02
Pour-on	11	0.22
Pour-on and hand dressing	1	0.02
Pour-on and traditional methods	1	0.02
Hand dressing	3	0.06
Injectable only	1	0.02
Traditional methods	2	0.04
No tick control	1	0.02

**Total**	**50**	**1**

## Discussion

The key findings have supported the suggestion by Mokantla et al. (2004) that long-mouthed ticks were responsible for significant damage to the scrotum of bulls on communal grazing. In the current study, the ticks present on the scrotum were identified as *A. hebraeum* and *H. rufipes*. Both species of long-mouthed ticks identified caused severe damage to the external genitalia of bulls (Walker et al. 2003). Abnormalities palpated and observed on the external genitalia were similar to those recorded by Mokantla et al. (2004). Scrotal lesions because of these ticks have also been described by other authors (Horak et al. 2017; Norval & Horak 2004; Spickett, Heyne & Williams 2011). This suggests that tick damage and high-level infestations may have played a role in reducing the semen quality. Damage to the scrotum could influence sperm production during the inflammation stage or cause chronic testicular induration. Damage to the prepuce caused by long-mouthed ticks could also prevent extrusion of the penis during copulation because of mechanical constriction or pain.

Only one of the farmers used plunge dipping, a preventative strategy that was once the standard for tick control as East Coast fever then was endemic in South Africa (Norval & Horak 2004). Because the disease was eradicated in 1954, dipping is no longer compulsory in the NWP and the plunge dips have disintegrated over time. The North West Provincial Department of Agriculture had built spray race dips in villages in the study area, which is probably why the majority of the respondents (*n* = 20) mentioned using spray dips. Traditional methods meant using old motor oil and Jeyes fluid, as described by Sekokotla (2004). However, using sprays and pour-on acaricides may be the reason why ticks feeding on the genital area proliferate, as these applications may not reach or are not applied to the ventral surface of the external genitalia. Injectable acaricides (Ivermectin 1% solution) are available from farmers’ co-operatives in the area, which is why they were a popular option (*n* = 11); however, they are relatively expensive and only last a few weeks. Ivermectin is not registered for use against the long-mouthed ticks observed in the study, although it is registered for the blue tick (*Rhipicephalus decoloratus*). It is possible that the high number of ticks on the external genitalia of bulls were because of acaricide resistance, but it is more likely that they were because of infrequent application. Mekonnen (2005) has suggested topical acaricide treatment every 14 days to control *A. hebraeum* and *H. rufipes* infestations. This suggested frequency of application would have been unlikely prior to the study, considering the dilapidated state of the crush pens, and was not observed during the study.

Another key finding was that the semen of only three (7.89%) of the 38 bulls tested was of good quality. All three of these bulls had been recently introduced to the study area and did not show the severe scrotal lesions observed in the other bulls. The bull/cow ratio (75/2398 or one bull per 32 cows) for the communal herds was fairly low, as DAFF (2018) suggest an appropriate ratio is one bull per 25 breeding cows on extensive grazing. Together with the poor semen quality in most (92.11%) of the 38 bulls tested, this was probably a contributory reason for the low calving rate. A bull with low semen quality may require more than one service to get a cow pregnant. The low calving rate is similar to the 37.86% previously reported in Jericho, which borders the study area (Mokantla et al. 2004). This situation is worse on communal grazing, as a bull may not be able to detect all of the cows in oestrus (Mokantla et al. 2004). The identification of two bulls that demonstrated seropositivity to *B. abortus* suggests that the prevalence of brucellosis in communal bulls was comparable to the prevalence of 3.7% found in communal cattle in the Gauteng Province (Njiro et al. 2011). Free vaccination of communal heifers (4–8 months) was done by the Directorate of Veterinary Services in the NWP, as mandated in terms of the *Animal Diseases Act*, 1984 (Act 35 of 1984) by DAFF. It is therefore probable that the two positive bulls were already infected when purchased. Brucellosis can affect bull fertility directly through damage to the testes, or indirectly through lowered calving rates when cows abort (OIE 2018).

The negative findings of the sheath scrapings in this study differed from the findings of Njiro et al. (2011), who previously recorded a prevalence of 2.1% of *T. foetus* in communal cattle in the Gauteng Province.

The mean SC in the bulls examined was 37.63 cm, above the minimum level recommended for breeding bulls. The daily production of high-quality sperm is linked to an acceptable SC because even if bulls with smaller SC produce semen of good quality, they will still have lower fertility because of reduced sperm per ejaculate (Chenoweth 1994; Chenoweth & McPherson 2016). However, a SC of 30 cm (the minimum measurement obtained) is not unusual in *Bos indicus* bulls over 24 months of age and on moderate-to-good nutrition (Entwhistle & Fordyce 2003).

Under circumstances of natural mating, the semen sample of a bull with 50% – 69% morphologically normal sperm can be regarded as satisfactory because there is a high probability of the bull being fertile (Chenoweth 1994; Chenoweth & McPherson 2016). In this study, 20 bulls (52%) showed 50% – 69% morphologically normal sperm (including live and dead spermatozoa). However, samples from these bulls were regarded as unsatisfactory because they failed to meet the minimum threshold of 30% progressive motility as suggested by Chenoweth and McPherson (2016) with 70% morphologically normal spermatozoa. Only three out of 38 bulls had morphologically normal sperm and demonstrated good motility according to breeding soundness criteria (Table 1).

Tick control strategies appeared to be erratic and in the absence of plunge dipping, it is likely that they were not effective in preventing the attachment of long-mouthed ticks in the scrotal and preputial area of range bulls. The lack of effective crush pens could also have influenced tick control as they were inadequate to restrain bulls for the application of acaricides.

These key findings have added to the existing knowledge about the value of long-mouthed tick control in rangeland and communal bulls and this could influence extension methods used by SVS to improve the annual calving rate. Veterinary extension methods could include empowering cattle owners to build effective crush pens and to use more targeted methods of tick control, linked to the seasonal activity of *H. rufipes* and *A. hebraeum* ticks in a specific communal area. The use of temporal and spatial determinants to control vectors are considered well-recognised tools in participatory epidemiology and risk analysis (Randolph 2000; Thrusfield et al. 2018).

The main strength of this study was that it was participatory. Farmers built their own crush pens with advice from the SVS. They were also empowered to recognise the types of ticks present and palpate the level of damage to the scrota of their bulls. This helped motivate them to allow fertility testing of the bulls and discuss methods of tick control with SVS officials. Another strength was that the communal farmers were involved in the regular counting of cows and calves and became more adept at monitoring their own herds. The relative risk for poor quality semen in bulls with scrotal lesions caused by long-mouthed ticks was very high, supporting a causal inference. When the calving rate is calculated annually in communal herds by counting calves, it links bull infertility to fewer calves in cows serviced by those bulls. It is suggested that perhaps the annual census approach to the number of calves in cattle herds, as used by DAFF, could be redefined using the term ‘annual calving rate’.

The main limitation of the study was the relatively small number of bulls tested. Five of these bulls had a SC that was suboptimal (Chenoweth 1994), which would decrease their fertility, whether or not tick damage was present. A confounding variable identified during the study was the use of acaricides, as owners of communal cattle did not keep records and relied on their memories. Another confounder was that on communal grazing land it is impossible to find out which bulls mated with specific cows and obviously more dominant (aggressive) bulls would mate more cows.

## Implications and recommendations

It is recommended that the SVS use the lessons learned from this study to encourage communal farmers to repair or rebuild their own crush pens using affordable materials. This would facilitate the application of effective acaricides using frequent spot acaricide treatment of the scrotum and prepuce, as well as the strategic application of injectable acaricides during the rainy season when the adult ticks attach. Further research is needed on the specific control of *Hyalomma* and *Amblyomma* ticks linked to their lifecycles, as well as their spatial and temporal epidemiology in communal grazing systems. Histopathology of the scrotal lesions would be very interesting as an aid to describing the pathophysiological reasons about why semen quality could be affected.
